# Diagnostic Potential of Systemic Eosinophil-Associated Cytokines and Growth Factors in IBD

**DOI:** 10.1155/2018/7265812

**Published:** 2018-07-29

**Authors:** Katarzyna Neubauer, Malgorzata Matusiewicz, Iwona Bednarz-Misa, Sabina Gorska, Andrzej Gamian, Malgorzata Krzystek-Korpacka

**Affiliations:** ^1^Department of Gastroenterology and Hepatology, Wroclaw Medical University, 50-556 Wroclaw, Poland; ^2^Department of Medical Biochemistry, Wroclaw Medical University, 50-368 Wroclaw, Poland; ^3^Laboratory of Medical Microbiology, Ludwik Hirszfeld Institute of Immunology and Experimental Therapy, Polish Academy of Sciences, 53-114 Wroclaw, Poland

## Abstract

Despite the acknowledged contribution of eosinophils to the disease pathogenesis, available data on cytokines closely related to the peripheral eosinophils in inflammatory bowel disease (IBD) are scattered. We assessed the concentrations of eosinophil-associated cytokines and growth factors in the group of 277 individuals (101 patients with Crohn's disease (CD), 77 with ulcerative colitis (UC), 16 with irritable bowel syndrome (IBS), and 83 healthy controls) and referred to IBD activity and the levels of hsCRP. As compared to IBS patients or healthy controls, patients with CD had significantly higher levels of IL5, IL8, IL12(p70), GM-CSF, and TNF*α* and patients with UC, the levels of eotaxin, IL4, IL5, IL8, IL12(p70), IL13, GM-CSF, and TNF*α* were also higher. As compared to CD patients, patients with UC had significantly higher levels of eotaxin, IL4, IL5, IL8, and IL1. In turn, the concentrations of hsCRP were significantly higher in CD than UC. Except for IL13, all cytokines and hsCRP positively correlated with CDAI. In UC, a positive correlation with MDAI was observed for hsCRP, GM-CSF, IL12(p70), and IFN*γ* and a negative one for IL8. The concentrations of hsCRP, GM-CSF, IFN*γ*, IL12(p70), and RANTES were higher in UC patients with active than inactive disease whereas those of IL8 and TNF*α* were significantly lower. Eotaxin, determined individually or in a panel with IFN*γ* and hsCRP, showed fair accuracy in differentiating CD from UC. If confirmed on a larger representation of IBS patients, IL8 might support differential diagnosis of organic and functional conditions of the bowel. GM-CSF, in turn, demonstrated to be an excellent indicator of bowel inflammation and may be taken into consideration as a noninvasive marker of mucosal healing. In summary, eosinophil-associated cytokines are elevated in IBD, more pronouncedly in UC, and may support the differential diagnosis of IBD and aid in monitoring of mucosal healing.

## 1. Introduction

Crohn's disease (CD) and ulcerative colitis (UC) are two main types of inflammatory bowel disease (IBD), a group of chronic inflammatory conditions of the bowel. IBD incidence is increasing worldwide, both in pediatric and adult populations [1, 2]. The disease is characterized by the wide spectrum of intestinal and extraintestinal complications. Pathogenesis of IBD is multifactorial and still not fully elucidated. Nevertheless, the consensus is that the crucial role is played by an impaired immune response to the gut microbiota, evoked by environmental triggers and/or genetic factors [3, 4]. The adaptive immunity seems to play the first fiddle in the pathogenesis of IBD [4]. However, the inflamed mucosa is infiltrated also by cytokine-rich innate immune cells [5] and their unceasing activation contributes to local and systemic inflammations [6].

IBD diagnostics and differential diagnostics pose a challenge for clinicians. IBD symptoms are nonspecific and overlapping. Establishing diagnosis requires a performance of series of laboratory, endoscopic, radiologic, and histologic tests. Those tests are expensive, often invasive, related to some risk, and poorly acceptable by patients. Biochemical markers currently used in clinical practice are insufficient and not recommended to serve as therapeutic targets [7]. Therefore, as a key part of the precision medicine strategy, the alternative, noninvasive biomarkers of IBD are intensively searched for [8, 9].

Eosinophils are acidophilic multifunctional granulocytes involved in inflammation and immunity that remain outside the mainstream research on IBD. However, they are a rich source of cytotoxic proteins, pro- and anti-inflammatory cytokines, chemokines, and growth factors and are likely to contribute to both inflammatory and regenerative phases of the disease. Accordingly, peripheral eosinophils of IBD patients are primed and preactivated. They display increased responsiveness, adhesiveness, migration, and degranulation [10, 11] and are characterized by upregulated secretion of their mediators, for example, eosinophilic cationic protein [12] or eotaxin [13, 14]. Peripheral eosinophilia is associated with less favorable outcome, that is, higher incidence of primary sclerosing cholangitis or need for surgical intervention [15]. Interestingly, prevalence of peripheral eosinophilia significantly differs between CD and UC. It has been reported to occur more often in UC patients, both in pediatric [16] and adult [15] populations. Locally, increased number and activation of eosinophils have been repeatedly observed in areas of active inflammation. Functionally, as evidenced in animal models of colitis, eosinophils seem to exert proinflammatory and promotility effects in IBD, thus contributing to diarrhea, tissue inflammation, and destruction, as well as fibrosis and formation of strictures. Additionally, they have been suggested to contribute to tissue repair and remodeling (reviewed in [17–19]). However, knowledge concerning eosinophils and quiescent bowel remains inconsistent.

Despite the acknowledged contribution of eosinophils to the disease pathogenesis, available data on cytokines closely related to the development and activity of peripheral eosinophils in IBD patients are either scattered or nonexistent. Herein, we assessed cytokines and growth factors crucial for eosinophil proliferation and differentiation (GM-CSF, IL5, and IL4), for their release from bone marrow (IL5), for their survival (IL5, IL13, GM-CSF, and IL4) and priming in circulation (IL5, GM-CSF, IL4, and TNF*α*), and for extravasation (IL4, IFN*γ*, TNF*α*, IL8, and eotaxin) and homing into the gut (eotaxin, RANTES, IL5, IL8, IL13, GM-CSF, and TNF*α*) as well as responsible for inducing production and secretion of their mediators (TNF*α*, IFN*γ*, IL-5, GM-CSF, and eotaxin) (reviewed in [10, 17, 20]). We also evaluated IL12 as a key inductor of eosinophil apoptosis and as a mediator of antieosinophil actions of glucocorticosteroids [10]. This paper focuses on the potential of eosinophil-associated cytokines and growth factors as biomarkers in IBD, in reference to high-sensitive C-reactive protein, an inflammatory marker commonly used in IBD clinics [8].

## 2. Materials and Methods

### 2.1. Study Population

Study population consisted of 277 individuals: 101 patients with Crohn's disease, 77 with ulcerative colitis, 16 with irritable bowel syndrome (IBS), and 83 healthy controls. IBD and IBS patients were recruited from the Department of Gastroenterology and Hepatology of Wroclaw Medical University, Poland. Individuals with indeterminate colitis or the coexistence of other severe systemic diseases, malignancies, liver diseases, or pregnancies were not included. The clinical activity of the disease was assessed using the Crohn's Disease Activity Index (CDAI) for CD and the Mayo Disease Activity Index (MDAI) for UC. Severity of bowel inflammation in UC patients was assessed using a Mayo endoscopic score. IBD patients were treated with 5′-aminosalicylate (5′-ASA) derivatives. Healthy controls were recruited from among students of our university and hospital staff or from among outpatients of Research, Science, and Educational Center of Dementia Diseases, Scinawa, Poland, diagnosed with mild cognitive disorders (no significant medical history) or from blood donors from the Regional Center for Blood Donation and Therapeutics in Wroclaw, Poland. Female to male ratio in study groups was as follows: 50/51 in CD, 34/43 in UC, 12/4 in IBS, and 38/45 in controls, *p* = 0.147. Age distribution was as follows (medians with 95% CI): 31 yrs (30–36.9) in CD, 40 yrs (33.8–45) in UC, 53 yrs (25–57) in IBS, and 35 yrs (18–87) in controls (*p* = 0.104).

### 2.2. Ethical Considerations

The study protocol was accepted by the Medical Ethics Committee of Wroclaw Medical University (KB-575/2011). The study was conducted in accordance with the Helsinki Declaration of 1975, as revised in 1983, and an informed consent was obtained from all patients.

### 2.3. Analytical Methods

Blood was drawn by venipuncture, following an overnight fast, allowed to clot for 30 minutes, and centrifuged (15 minutes, 720 ×g). Resulting sera were collected, aliquoted, and kept frozen at −80° until examination. We applied a flow cytometry-based method utilizing magnetic microspheres conjugated with monoclonal antibodies (Luminex xMAP® Technology) to measure the concentrations of eotaxin, GM-CSF, IFN*γ*, IL4, IL5, IL8, IL12(p70), IL13, RANTES, and TNF*α*. Analytes were measured in duplicate using Bio-Plex Pro™ human cytokine, chemokine, and growth factor magnetic bead-based assays according to the instructions provided by the manufacturer but with a lower serum dilution factor and the Bio-Plex 200 platform with HRF (Bio-Rad, USA). Standard curves were drawn using 5-PL logistic regression, and the data were analyzed using Bio-Plex Manager 6.0 software. Data on high-sensitive C-reactive protein (hsCRP), measured using an enhanced immunoturbidimetric method with the CRPex-HS CRP test (Good Biotech Corp., Taichung, Taiwan), were retrieved from our earlier study on this population (available exclusively for IBD patients).

### 2.4. Statistical Analysis

Normality of data distribution was tested using the *χ*^2^ test and homogeneity of variances using Levene's test. Data were log-transformed if necessary and presented as geometric means or medians accompanied by 95% confidence interval. Multigroup comparisons were conducted using one-way ANOVA or the Kruskal-Wallis *H* test. Two-group comparisons were conducted using *t*-test for independent samples (with Welch correction if appropriate) or the Mann–Whitney *U* test. Correlation analysis with the disease activity scores was conducted using the Spearman test. Frequency analysis was conducted using the *χ*^2^ test or Fisher's exact test. Receiver operating characteristic (ROC) curve analysis was conducted to evaluate the strength of observed associations and the suitability of analytes as disease markers. A backward stepwise method of logistic regression, followed by the Hosmer and Lemeshow goodness-of-fit test and ROC analysis, was used to assess the discriminative power of combined marker evaluation. Model building was preceded by the analysis of linearity, collinearity, and interactions of candidate variables. To validate the final model, we applied the *V*-fold cross-validation. *Z* statistics was used to evaluate the significance of difference between area under ROC curves (AUCs; measure of test accuracy) obtained for training and validation sets. All calculated probabilities were two-tailed, and *p* values ≤ 0.05 were considered statistically significant. The statistical analysis was conducted using MedCalc statistical software version 15.8 (MedCalc Software bvba, Ostend, Belgium; https://www.medcalc.org; 2015) and Medical Bundle ver. 3.0., an add-in to Statistica 12 PL (StatSoft Polska Sp. z o.o. 2016; http://www.statsoft.pl).

## 3. Results

### 3.1. Eosinophil-Associated Cytokines in Bowel Diseases

**Figure 1:**
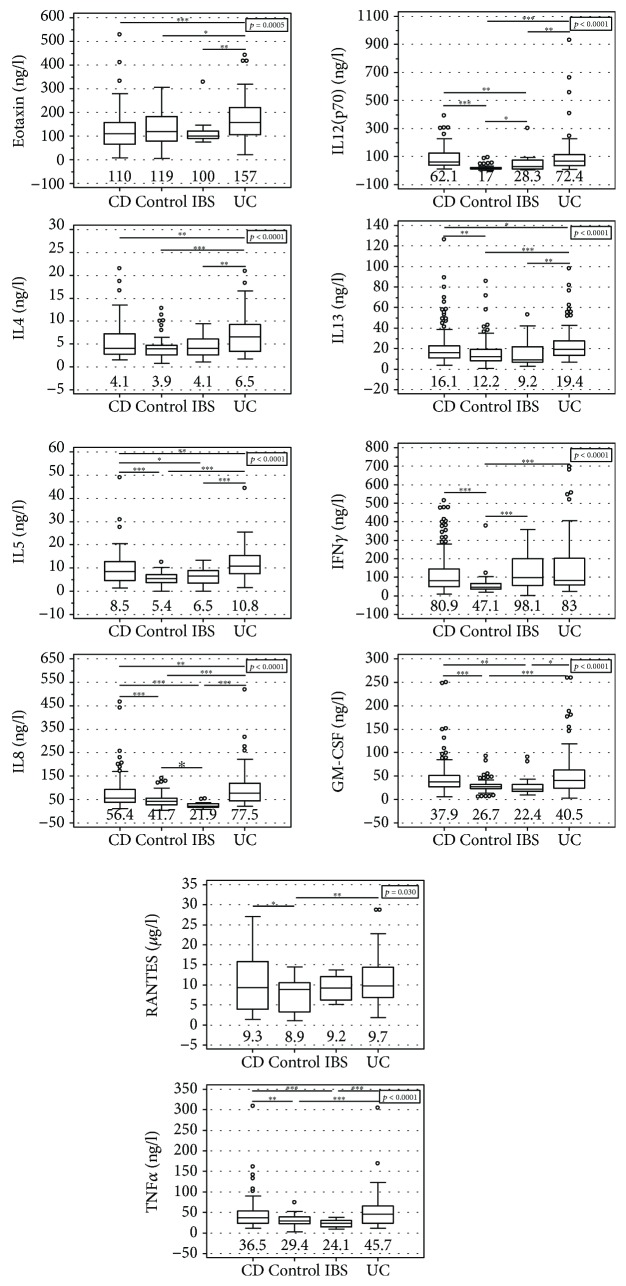
Systemic eosinophil-associated cytokines in patients with inflammatory bowel disease or irritable bowel syndrome and healthy controls. Data presented as medians (bar within box) with interquartile range (box) and 91% CI (whiskers) and analyzed with Kruskal-Wallis *H* test. Open circles mark outlying observations. Horizontal bars with asterisks indicate statistically significant between-group differences: ^∗^*p* < 0.05, ^∗∗^*p* < 0.01, and ^∗∗∗^*p* < 0.001. CD: Crohn's disease; CONTROL: controls; IBS: irritable bowel syndrome; UC: ulcerative colitis.

Patients with UC had significantly higher levels of eotaxin, IL4, IL5, IL8, and IL13 than patients with CD or IBS and healthy controls. They also had higher levels of TNF*α*, GM-CSF, and IL12(p70) than IBS patients and healthy controls but not CD patients. The levels of RANTES and IFN*γ* were higher in UC patients but only in comparison to healthy controls (Figure 1).

Patients with CD had significantly higher levels of IL5, IL8, IL12(p70), GM-CSF, and TNF*α* than IBS patients and healthy controls and higher levels of IL13, IFN*γ*, and RANTES as compared to healthy controls (Figure 1).

Patients with IBS had significantly higher levels of IL12(p70) and IFN*γ* than healthy controls but lower levels of IL8 (Figure 1).

The concentrations of hsCRP in IBD patients were higher in CD than UC: 30.7 mg/l (25.9–55.8) and 6.1 mg/l (3.2–17.8), *p* < 0.0001, respectively.

### 3.2. Cytokine Relation to IBD Activity

From among evaluated cytokines, IL12(p70) was the sole cytokine that differed significantly between CD patients with active and inactive disease: 72.7 pg/ml (63–84) and 74.2 pg/ml (29–78), *p* = 0.034, respectively.

In UC, active disease was associated with more pronouncedly elevated levels of GM-CSF (47 pg/ml (39–57) versus 23.3 pg/ml (15–36), *p* < 0.001), IFN*γ* (95 pg/ml (83–154) versus 65 pg/ml (57–81), *p* = 0.001), IL12(p70) (81.8 pg/ml (66–102) versus 42.4 pg/ml (27–66), *p* = 0.003), and RANTES (10.1 ng/ml (9–13) versus 8.1 ng/ml (6–10), *p* = 0.053) than inactive disease. However, active UC was accompanied by lower levels of IL8 (70 pg/ml (58–85) versus 103 pg/ml (82–130), *p* = 0.021) and TNF*α* (38.5 pg/ml (32–46) versus 55.5 pg/ml (46–67), *p* = 0.007).

Except for IL13, all evaluated cytokines and growth factors weakly to moderately correlated with CDAI. In turn, only IL13 and GM-CSF displayed a positive association with MDAI (Table 1).

Of the examined cytokines, GM-CSF, IL12(p70), and IFN*γ* positively correlated with the degree of endoscopic inflammation in UC patients (Table 1). IL8 displayed a negative correlation due to high cytokine levels in patients without inflammation. When the analysis was restricted to patients with inflamed mucosa, the association tended to be positive (*p* = 0.107).

The levels of hsCRP were significantly higher in active than inactive CD: 38.6 mg/l (27–65) and 1.74 mg/l (0.7–21), *p* = 0.002, respectively. They were also higher in active than inactive UC: 18 mg/l (5.5–22.6) and 1.75 mg/l (0.3–3.8), *p* < 0.001, respectively. There was a positive correlation between hsCRP and the indices of clinical activity of CD and UC as well as endoscopic inflammation in UC (Table 1).

### 3.3. Eosinophil-Associated Cytokines as Differential Markers in IBD

**Figure 2:**
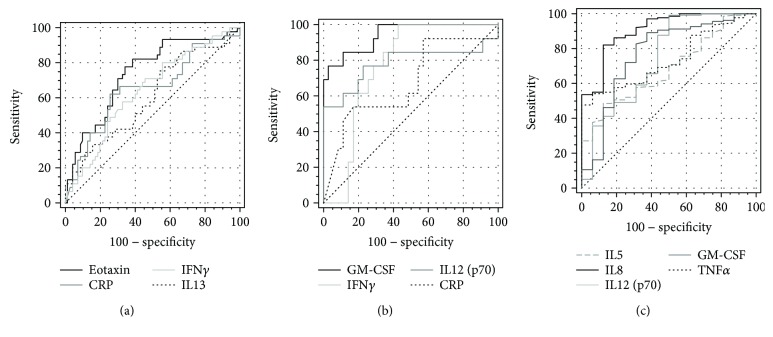
Eosinophil-associated cytokines as biomarkers in IBD-receiver operating characteristic (ROC) curves. (a) Eosinophil-associated cytokines as markers differentiating active UC and CD; (b) eosinophil-associated cytokines as mucosal healing markers; (c) eosinophil-associated cytokines as markers differentiating active IBD and IBS.

Symptomatology of UC and CD may sometimes overlap, rendering both IBD types difficult to distinguish. Moreover, there is still a group of patients with unclassified IBD, in whom the definitive distinction between UC and CD is not possible despite of all recommended tests [21]. Of the evaluated eosinophil-associated cytokines, eotaxin (149.3 pg/ml (124.8–178.7) versus 103.4 pg/ml (91.6–116.7), *p* < 0.001), IFN*γ* (127.6 pg/ml (100.4–162.3) versus 94.2 pg/ml (78.2–113.4), *p* = 0.046), and IL13 (22.1 pg/ml (18.1–27.1) versus 16.9 pg/ml (14–6.19.7), *p* = 0.035) were more pronouncedly elevated in active UC than CD. In turn, hsCRP was significantly higher in active CD than UC (26.6 mg/l (17.2–41.2) versus 8.9 mg/l (4.3–18.4), *p* = 0.011). We applied ROC analysis to evaluate the suitability of these cytokines and hsCRP as individual differential markers (Figure 2(a)). As depicted in Table 2, eotaxin displayed superior accuracy as a marker of active UC, followed by hsCRP as a marker of active CD.

In order to discern the independent predictors of the disease type and evaluate the power of their combined assessment, we applied logistic regression. All cytokines, the levels of which significantly differed between active UC and CD, were selected as potential explanatory variables, and active CD was coded as 0 while active UC coded as 1. Data on CRP levels were unavailable for a few patients; thus, the analysis was limited to 115 patients with active IBD (45 with UC and 70 with CD). First, a univariate regression analysis was conducted for each variable and all variables were found to significantly affect the dependent variable. Next, candidate variables were tested for linearity, collinearity, and interactions. The model was built with eotaxin, IL13, IFN*γ*, and hsCRP (ratio of analyzed variables to cases was 29), and a backward stepwise method was used. Eotaxin (*b* = 3.3, *p* = 0.002; OR = 27 (4–210); constant = −9, *p* < 0.001), hsCRP (*b* = −0.54, *p* = 0.020; OR = 0.58 (0.37–0.92)), and IFN*γ* (*b* = 1.1, *p* = 0.069; OR = 2.95 (1–9.5)) contributed to model's predictive power and were retained in the final model whereas IL13 was not included. The overall fit of the three-parameter model was *χ*^2^ = 25.8, *p* < 0.0001, and the results of the Hosmer and Lemeshow test were *χ*^2^ = 5.3, *p* = 0.731 indicating a good logistic regression model fit. The model predictive power was fair (Table 2) and allowed for a correct classification of 72.2% of cases; however, the model's pseudo *R*-squared was rather low: Nagelkerke *R*^2^ = 0.273. The accuracy of a validated model (*V*-fold cross-validation) was comparable (Table 2). Lack of significant difference in accuracy between training and validation sets suggests that the predictive power of the model was not overestimated.

### 3.4. Eosinophil-Associated Cytokines as Negative Markers of Mucosal Healing (MH) in UC

Data on endoscopic findings were available for UC patients, among whom 16 had no signs of active bowel inflammation (coded as 1 for the purpose of ROC analysis and logistic regression) and 37 had an ongoing inflammation (coded as 0). Of patients with active inflammation, 10 were assigned Mayo endoscopic score 1, 15 score 2, and 12 score 3.

GM-CSF, IFN*γ*, and IL12(p70) as well as hsCRP were significantly correlated with Mayo endoscopic subscore (Table 1). Individually, the accuracy of GM-CSF as the marker of mucosal healing was superior to those of other cytokines and hsCRP (Figure 2(b), Table 3).

Logistic regression (backward stepwise methods) was applied to discern the independent predictors of mucosal healing and to evaluate the power of potential multimarker assays. GM-CSF, IFN*γ*, IL12(p70), and hsCRP were tested as potential explanatory variables. However, they were found to be interrelated; GM-CSF was positively correlated with hsCRP (*r* = 0.56, *p* < 0.00001), IL12(p70) (*r* = 0.404, *p* = 0.003), and IFN*γ* (*r* = 0.035, *p* = 0.011). Also, hsCRP was correlated with IFN*γ* (*r* = 0.36, *p* = 0.014). As such, we attempted to build three separate models: one exclusively with GM-CSF, second containing IFN*γ* and IL12(p70) combined together, and third with hsCRP and IL12(p70) combined together. However, testing for linearity and collinearity excluded two latter combinations of variables. At the end, only a model with GM-CSF was built. Regression coefficient for GM-CSF was *b* = −7.7, *p* < 0.001; OR was 0.0005 (0–0.0335); constant = 10.33, *p* < 0.001. The overall model fit was *χ*^2^ = 30.5, *p* < 0.00001, and the results of the Hosmer and Lemeshow test were *χ*^2^ = 6.8, *p* = 0.658 indicating a good logistic regression model fit. The model predictive power was excellent (Table 3) and allowed for a correct classification of 87% of cases. The model's pseudo *R*-squared was high as well: Nagelkerke *R*^2^ = 0.619. The accuracy of the *V*-fold cross-validated model was comparable proving the model predictive power not to be overestimated (Table 3).

### 3.5. Eosinophil-Associated Cytokines as Markers Differentiating between IBD and IBS

Functional bowel disorders like IBS display a similar set of symptoms to IBD making their differential diagnosis difficult. Herein, we evaluated the eosinophil-associated cytokines as potential differential markers.

As depicted in Figure 1, IL5, IL8, IL12(p70), TNF*α*, and GM-CSF were significantly higher in both CD and UC than in IBS. Of these, IL8 had the highest AUC and Youden index. At optimal cut-off values, IL5 and TNF*α* displayed superior specificity, IL12(p70) and GM-CSF displayed superior sensitivity, and the sensitivity and specificity of IL8 was equally high (Figure 2(c), Table 4).

Logistic regression was applied to discern the independent indicators of active IBD (coded as 1 and IBS coded as 0). Candidate variables (IL5, IL8, IL12(p70), GM-CSF, and TNF*α*) were tested for linearity, collinearity, and interactions. IL5, IL8, and TNF*α* occurred to be interrelated with the following correlation coefficients: *r* = 0.74 for IL5 and IL8, *r* = 0.6 for IL5 and TNF*α*, and *r* = 0.62 for IL8 and TNF*α*. As such, three separate models were tested: each model included GM-CSF and IL12(p70) and either IL5, IL8, or TNF*α*. Of these, the model with IL8 (IL8, GM-CSF, and IL12(p70)) displayed the best fit. However, following the analysis of variable contribution to the model's predictive power, exclusively IL8 was retained. Regression coefficient for IL8 was *b* = 4.49, *p* < 0.0001; OR was 89.4 (11.8–679); constant = −13.9, *p* < 0.0001. The overall model fit was *χ*^2^ = 43.3, *p* < 0.00001, and the results of the Hosmer and Lemeshow test were *χ*^2^ = 5.3, *p* = 0.727 indicating a good logistic regression model fit. The model predictive power was excellent (Table 4) and allowed for a correct classification of 93% of cases. The model's pseudo *R*-squared (Nagelkerke *R*^2^) was 0.502. AUC obtained after *V*-fold cross-validation was slightly lower, but there was no significant difference compare to AUC of a training set proving the model predictive power not to be overestimated (Table 4).

## 4. Discussion

Eosinophils are gaining an increasing attention as the cells of unique properties among leukocytes, which can damage or repair surrounding tissue and modulate the activity of immune cells [22]. Accordingly, the infiltration of lamina propria by eosinophils has been shown to be an early histological marker of UC [23] and a predictor of clinical relapse [24] as well as poor response of UC patients to medical therapy [25]. Recently, local eosinophilia has been reported to be related to the stricturing phenotype of CD [26]. Peripheral eosinophilia has been observed in IBD as well and linked to poor prognosis [15]. Peripheral eosinophils isolated from IBD patients have been characterized as primed, displaying increased responsiveness, adhesiveness, migration, and degranulation [10, 11]. Still, little is known on cytokines involved in managing growth, distribution, and priming of eosinophils as well as in aiding their homing into the gut. Except for eotaxin, which has become a novel therapeutic target in UC [27], eosinophil-associated cytokines, such as GM-CSF, IL3, IL5, and IL13, have not been comprehensively analyzed in the context of IBD. Available data are scanty, mostly derived from the analysis of small cohorts, and obtained exclusively from multiplexed analyses intended for screening purposes, in which cytokines less abounded in sera frequently balance at the verge of lower detection limits of the tests, making their evaluation problematic [28–30]. Herein, the dilution of our samples was adjusted to enable credible analysis of these analytes. It allowed us to show that compared to healthy individuals, IBD patients had significantly higher concentrations of all evaluated eosinophil-associated cytokines and growth factors, although the elevation of eotaxin and IL4 was observed exclusively in UC patients.

Differential diagnosis of UC and CD might be challenging [21]. In this respect, it is easy to perform and noninvasive cytokine assays would be welcomed to assist clinicians in the diagnostic process. In line with higher prevalence of local [31] and peripheral eosinophilia [16] in UC than CD, we found several of eosinophil-associated cytokines to be more pronouncedly elevated in UC. We evaluated suitability of eotaxin, IL13, IFN*γ*, and hsCRP as differential markers assessed individually as well as a multicytokine panel. Individually, eotaxin performance was fair and superior to other markers. Eotaxin is a critical chemoattractant specific for eosinophils, and an elevation of its circulating concentrations in IBD has already been reported by several groups [13, 14, 28, 29, 32]. However, previous comparative analyses of eotaxin in CD and UC have been conducted exclusively in small cohorts and yielded contradictory results [13, 14, 28, 32]. The addition of IFN*γ* and hsCRP improved the discriminative power of the assay as compared to sole determination of eotaxin but not markedly. Of note, Iboshi et al. [33] found the expression of IFN*γ* to be more accentuated in UC than in CD.

Chronic bowel inflammation is a hallmark of IBD. The assessment of its severity is imperative for the determination of the disease activity and establishment of a therapeutic approach. It became particularly crucial in view of the recent change of priorities in IBD therapy from control of symptoms to the restriction of inflammation [1]. Mucosal healing (MH), currently assessed exclusively by endoscopy, is related to more favorable clinical outcomes and has lately become one of the therapeutic endpoints in clinical trials. However, clinical trials have shown a poor correlation between clinical symptoms and endoscopy. It is estimated that almost half of CD patients with clinical remission are still presenting with endoscopic features of active disease whereas almost 40% of patients with inactive disease in endoscopy are displaying clinical symptoms of the disease [34]. Since clinical indices are not sufficient, biomarkers, which could be applied in MH evaluation, as well as in monitoring the disease course, predicting its relapse, and recognizing flare, are intensively sought after [34–36]. Herein, hsCRP, GM-CSF, IFN*γ*, and IL12(p70) were significantly and positively correlated with the degree of bowel inflammation, expressed as Mayo endoscopic subscore. Of these, GM-CSF has emerged as a superior MH marker in UC. GM-CSF displayed high accuracy, falling within the range typical for biomarkers applied in clinical practice (85–95%). Importantly, GM-CSF accuracy remained high following its validation. Taking into account the invasiveness and a risk for complications associated with endoscopy, serum-based MH markers such as GM-CSF might help to assist in the selection of patients and limit the need for invasive examinations. GM-CSF has been the only cytokine other than eotaxin significantly elevated in sera of UC patients in Coburn et al.'s study [29]. GM-CSF controls proliferation and differentiation if eosinophils in the bone marrow provide survival signals and induce the release of their granule proteins. GM-CSF is also responsible for eosinophil priming in circulation and acts as their chemoattractant [20, 37]. However, as reviewed by Han et al. [38], data available on GM-CSF functionality in the bowel are equivocal. A beneficial role has been attributed to this growth factor in the small intestine, where it might contribute to maintaining intestinal barrier function, likely through stimulating proliferation of intestinal epithelial cells (IECs). Accordingly, no difference in GM-CSF expression has been noted between inflamed and noninflamed IECs. On the other hand, colonic mucosa has been shown to overexpress GM-CSF when inflamed or cancerous and the growth factor had no effect on colonic cell proliferation. Despite unresolved issue of GM-CSF function in IBD, growth factor-based therapies have been attempted and yielded promising results. In animal models of colitis, GM-CSF application has resulted in an improvement of clinical symptoms and histological findings as well as in accelerated ulcer healing. In human CD, a therapy with recombinant GM-CSF has lessened the disease severity (reviewed in [38]). In the light of these findings, our observation on GM-CSF increase in IBD patients might represent a protective mechanism. However, healing capabilities of GM-CSF might be counteracted by concomitant elevation in the levels of GM-CSF autoantibodies. Their presence in IBD patients and the association with a more aggressive disease phenotype have been previously reported and implied in functional GM-CSF deficiency caused by antibody interference with GM-CSF binding to its receptor [39, 40].

It is estimated that the endoscopy in about half of adult and 70% of pediatric patients presenting with symptoms indicative of IBD yields negative results. These patients are subsequently diagnosed with functional bowel disorders, for example, IBS, the condition which share many clinical symptoms with IBD. In this respect, a reliable but less invasive differential tool is needed [41]. From among eosinophil-associated cytokines analyzed by us, IL5, IL8, IL12(p70), TNF*α*, and GM-CSF were significantly higher in both CD and UC than in IBS. Of these, IL8 had superior accuracy in differentiating IBS and IBD and allowed for a correct classification of 93% of patients. IL8 is a cytokine which is the most loosely associated with eosinophils. Although secreted by these leukocytes and acting as their chemoattractant [20], IL8 is primarily responsible for neutrophil trafficking. Correspondingly, another neutrophil-associated protein, namely, calprotectin, has been claimed as having adequate sensitivity and specificity to assist in differentiating IBD from IBS. Even though IL8 herein displayed an excellent accuracy, validated using *V*-fold cross-validation technique, our results should be interpreted with care and treated as indicative only because of the small number of patients with IBS included in our cohort.

## 5. Conclusions

Our results obtained on a large cohort of patients showed that the concentrations of circulating eosinophil-associated cytokines are elevated in IBD and that this elevation is more accentuated in UC. Evaluated as prospective differential markers, eotaxin, individually and in a panel with IFN*γ* and hsCRP, showed fair accuracy in differentiating CD from UC. IL8, if confirmed on a larger representation of IBS patients, might, in turn, support differential diagnosis of organic (IBD) and functional (IBS) conditions of the bowel. GM-CSF demonstrated to be an excellent indicator of bowel inflammation and may be taken into consideration as a noninvasive marker of mucosal healing.

## Figures and Tables

**Table 1:** Correlation between eosinophil-associated cytokines, chemokines, and growth factors and disease activity.

Cytokine	Clinical activity		Endoscopic activity in UC
	CD (CDAI)	UC (Mayo)	
Eotaxin	0.28, *p* = 0.009	ns	ns
GM-CSF	0.30, *p* = 0.005	0.33, *p* = 0.003	0.56, *p* < 0.0001
IL4	0.35, *p* = 0.001	ns	ns
IL5	0.38, *p* < 0.001	ns	ns
IL8	0.38, *p* < 0.001	ns	−0.38, *p* = 0.005
IL12(p70)	0.35, *p* < 0.001	0.511, *p* < 0.0001	0.49, *p* < 0.001
IL13	ns	ns	ns
IFN*γ*	0.25, *p* = 0.019	0.45, *p* < 0.0001	0.55, *p* < 0.0001
RANTES	0.24, *p* = 0.028	ns	ns
TNF*α*	0.22, *p* = 0.037	ns	ns
hsCRP	0.40, *p* < 0.001	0.66, *p* < 0.0001	0.46, *p* < 0.001

ns: not significant.

**(a):** 

Marker	AUC (95% CI), *p*_AUC = 0.5_	Cut-off	Sens.&Spec.	*J* index
Eotaxin	0.708 (0.62–0.78), *p* < 0.0001	>138 pg/ml	71 and 68%	0.391
IL13	0.604 (0.52–0.69), *p* = 0.032	>20.8 pg/ml	51 and 69%	0.203
IFN*γ*	0.594 (0.51–0.68), *p* = 0.054	>64.5 pg/ml	76 and 44%	0.199
hsCRP^∗^	0.657 (0.57–0.74), *p* = 0.003	≥19.9 mg/l	74 and 60%	0.347

**(b):** 

Panel	AUC (95% CI)		Difference
	Learning set	Validation set	*z* statistics, *p*
Eotaxin, hsCRP, IFN*γ*	0.78 (0.7–0.87)	0.73 (0.63–0.82)	0. 818, *p* = 0.414

^∗^hsCRP was tested as an indicator of CD. AUC: area under ROC curve; Sens.&Spec.: sensitivity and specificity; *J* index: Youden index.

**(a):** 

Marker	AUC (95% CI), *p*_AUC = 0.5_	Cut-off	Sens.&Spec.	*J* index
GM-CSF	0.907 (0.80–0.97), *p* < 0.001	≤16.7 pg/ml	69 and 97%	0.661
IFN*γ*	0.782 (0.65–0.88), *p* < 0.001	≤83.2 pg/ml	100 and 60%	0.595
IL12(p70)	0.709 (0.57–0.83), *p* = 0.033	≤21.6 pg/ml	50 and 100%	0.500
hsCRP	0.673 (0.52–0.80), *p* < 0.001	≤0.5 mg/l	54 and 83%	0.367

**(b):** 

Panel	AUC (95% CI), *p*_AUC = 0.5_		Difference
	Learning set	Validation set	*z* statistics, *p*
GM-CSF	0.907 (0.80–0.97)	0.884 (0.78–0.99)	0.335, *p* = 0.738

AUC: area under ROC curve; Sens.&Spec.: sensitivity and specificity; *J* index: Youden index.

**(a):** 

Marker	AUC (95% CI), *p*_AUC = 0.5_	Cut-off	Sens.&Spec.	*J* index
IL5	0.69 (0.61–0.76), *p* = 0.003	>9.68 pg/ml	48.6 and 87.5%	0.361
IL8	0.91 (0.85–0.95), *p* < 0.0001	>36.8 pg/ml	82.1 and 87.5%	0.696
IL12(p70)	0.75 (0.67–0.81), *p* = 0.002	>22.6 pg/ml	99.3 and 50%	0.493
TNF*α*	0.74 (0.66–0.81), *p* < 0.0001	>34 pg/ml	54.3 and 93.8%	0.480
GM-CSF	0.77 (0.7–0.84), *p* < 0.001	>24 pg/ml	89.3 and 62.5%	0.518

**(b):** 

Panel	AUC (95% CI), *p*_AUC = 0.5_		Difference
	Learning set	Validation set	*z* statistics, *p*
IL8	0.91 (0.85–0.95), *p* < 0.0001	0.87 (0.76–0.98), *p* < 0.0001	0.503, *p* = 0.615

AUC: area under ROC curve; Sens.&Spec.: sensitivity and specificity; *J* index: Youden index.

## Data Availability

The data used to support the findings of this study are included within the article.
